# Supplementary material for: Fast grassland recovery from viable propagules after reintroducing traditional mowing management on a steep slope
Source: PeerJ. 2024 Jun 5;12:e17487. doi: 10.7717/peerj.17487 (PMC11162179; doi:10.7717/peerj.17487)
Supplement: Supplemental Information 1 — A, annual; P, perennial herb; W, woody species. Species with a frequency ≥ 0.2 in at least 1 year are shown. Increases and decrease were defined when differences in frequency between years were >0.2. Several individuals of Cirsium spp. died before identification, so frequencies for C. oligophyllum and C. japonicum would be underestimated. *All the seedlings of Carex ssp. were unidentified to the species level. [file peerj-12-17487-s001.docx]

**Supplementary Table S1** Monthly precipitation and temperature in the study area in 2008, 2018, 2019 and 2020 and the average (30 years period, between 1981-2010)

a) precipitation (mm)

|  | average | 2008 | 2018 | 2019 | 2020 |
| --- | --- | --- | --- | --- | --- |
| Jan | 54.1 | 13.0 | 38.0 | 12.5 | 107.0 |
| Feb | 46.1 | 36.0 | 11.5 | 23.5 | 9.0 |
| Mar | 102.9 | 75.5 | 235.5 | 119.5 | 101.0 |
| Apr | 114.1 | 238.5 | 64.0 | 86.0 | 257.0 |
| May | 124.7 | 316.5 | 123.5 | 128.5 | 67.0 |
| Jun | 172.0 | 228.0 | 112.0 | 211.0 | 217.5 |
| Jul | 188.3 | 78.0 | 139.0 | 212.0 | 350.0 |
| Aug | 212.5 | 666.5 | 181.5 | 200.0 | 37.0 |
| Sep | 264.4 | 223.5 | 399.5 | 152.5 | 233.5 |
| Oct | 230.8 | 91.5 | 66.0 | 673.0 | 213.0 |
| Nov | 80.1 | 70.5 | 21.0 | 97.0 | 11.5 |
| Dec | 53.1 | 65.5 | 44.5 | 50.5 | 1.0 |

b) temperature (°C)

|  | average | 2008 | 2018 | 2019 | 2020 |
| --- | --- | --- | --- | --- | --- |
| Jan | 3.4 | 3.4 | 2.6 | 3.3 | 5.6 |
| Feb | 4.5 | 3.1 | 3.9 | 5.6 | 6.5 |
| Mar | 8.0 | 9.1 | 10.3 | 9.3 | 9.6 |
| Apr | 13.3 | 13.4 | 15.9 | 12.7 | 11.9 |
| May | 18.1 | 17.2 | 19.1 | 18.9 | 18.9 |
| Jun | 21.4 | 20.3 | 22.1 | 21.2 | 22.8 |
| Jul | 25.4 | 26.1 | 28.1 | 23.8 | 23.5 |
| Aug | 26.4 | 25.5 | 27.6 | 27.6 | 28.6 |
| Sep | 22.6 | 22.6 | 22.1 | 24.2 | 23.3 |
| Oct | 16.9 | 17.5 | 17.8 | 18.5 | 16.3 |
| Nov | 11.1 | 10.9 | 12.5 | 11.7 | 12.3 |
| Dec | 5.9 | 7.1 | 6.7 | 6.9 | 5.9 |
