# Supplementary material for: Fast grassland recovery from viable propagules after reintroducing traditional mowing management on a steep slope
Source: PeerJ. 2024 Jun 5;12:e17487. doi: 10.7717/peerj.17487 (PMC11162179; doi:10.7717/peerj.17487)
Supplement: Supplemental Information 2 — Mean values ± SD are shown. [file peerj-12-17487-s002.docx]

**Supplementary Table S2** Changes in frequency (number of plots in which species was found / number of surveyed plots) of major species in emergent vegetation (2008, 2018, 2019, and 2020), seedlings (2019 and 2020), and vegetative sprouts (2019)

|  |  |  |  | Life | Other | 2008 | 2018 | 2019 | | |  | 2020 | |
| --- | --- | --- | --- | --- | --- | --- | --- | --- | --- | --- | --- | --- | --- |
| Group | | | Species | history | trait |  |  |  | (seedling) | (sprout) |  |  | (seedling) |
| a) | Dominant matrix forming grasses | | |  |  |  |  |  |  |  |  |  |  |
|  |  |  | *Miscanthus sinensis* | P | Grassland | 0.7 | 0.8 | 1.0 | (0.5) | (1.0) |  | 0.9 | (0.0) |
|  |  |  | *Pleioblastus chino* | P | Grassland | 0.7 | 0.5 | 0.8 | (0.0) | (0.8) |  | 0.8 | (0.0) |
|  |  |  | *Imperata cylindrica.* var. *koenigii* | P | Grassland | 0.9 | 0.2 | 0.1 | (0.0) | (0.1) |  | 0.5 | (0.0) |
|  |  |  | *Arundinella hirta* | P | Grassland | 0.5 | 0.0 | 0.1 | (0.0) | (0.0) |  | 0.2 | (0.0) |
|  |  | Average | |  |  | 0.70 | 0.38 | 0.50 | (0.13) | (0.48) |  | 0.60 | (0.00) |
| b) | Middle-statured subordinate perennial forbs | | |  |  |  |  |  |  |  |  |  |  |
|  |  |  | *Gentiana scabra* var. *buergeri* | P | Grassland | 0.7 | 0.3 | 0.1 | (0.0) | (0.1) |  | 0.2 | (0.1) |
|  |  |  | *Solidago virgaurea* subsp. *asiatica* | P | Grassland | 0.3 | 0.0 | 0.0 | (0.0) | (0.0) |  | 0.0 | (0.1) |
|  |  |  | *Cirsium oligophyllum* | P | Grassland | 0.8 | 0.2 | 0.6 | (0.5)* | (0.3) |  | 0.7 | (0.3) |
|  |  |  | *Cirsium japonicum* | P | Grassland | 0.7 | 0.3 | 0.6 | (0.4)* | (0.4) |  | 0.8 | (0.2) |
|  |  |  | *Thalictrum minus* var. *hypoleucum* | P | Grassland | 0.1 | 0.4 | 0.7 | (0.8) | (0.2) |  | 0.7 | (0.8) |
|  |  |  | *Euphorbia lasiocaula* | P | Grassland | 0.3 | 0.6 | 0.7 | (0.7) | (0.7) |  | 0.7 | (0.7) |
|  |  |  | *Sanguisorba officinalis* | P | Grassland | 0.6 | 0.4 | 0.7 | (0.1) | (0.7) |  | 0.7 | (0.0) |
|  |  | Average | |  |  | 0.50 | 0.31 | 0.49 | (0.36) | (0.34) |  | 0.54 | (0.31) |
| c) | Small-statured subordinate perennial forbs | | |  |  |  |  |  |  |  |  |  |  |
|  |  |  | *Potentilla freyniana* | P | Small, Grassland | 0.9 | 0.1 | 0.4 | (0.3) | (0.4) |  | 0.5 | (0.7) |
|  |  |  | *Barnardia japonica* | P | Small, Grassland | 0.0 | 0.0 | 1.0 | (0.3) | (1.0) |  | 0.2 | (0.1) |
|  |  |  | *Polygala japonica* | P | Small, Grassland | 0.2 | 0.0 | 0.0 | (0.4) | (0.0) |  | 0.4 | (0.1) |
|  |  | Average | |  |  | 0.37 | 0.03 | 0.47 | (0.33) | (0.47) |  | 0.37 | (0.30) |
| d) | Other small-statured perennials | | |  |  |  |  |  |  |  |  |  |  |
|  |  |  | *Viola grypoceras* var. *grypoceras* | P | Small | 0.5 | 0.1 | 0.1 | (0.1) | (0.1) |  | 0.1 | (0.0) |
|  |  |  | *Galium gracilens* | P | Small | 0.4 | 0.0 | 0.1 | (0.0) | (0.1) |  | 0.1 | (0.0) |
|  |  |  | *Lysimachia japonica* | P | Small | 0.5 | 0.0 | 0.0 | (0.6) | (0.0) |  | 0.5 | (0.9) |
|  |  |  | *Oxalis corniculata* | P | Small | 0.7 | 0.0 | 0.0 | (0.6) | (0.0) |  | 0.4 | (0.1) |
|  |  |  | *Akebia trifoliata* | P | Small | 0.5 | 0.2 | 0.4 | (0.0) | (0.4) |  | 0.3 | (0.0) |
|  |  |  | *Ajuga decumbens* | P | Small | 0.1 | 0.0 | 0.0 | (0.4) | (0.0) |  | 0.0 | (0.1) |
|  |  | Average | |  |  | 0.45 | 0.05 | 0.10 | (0.28) | (0.10) |  | 0.23 | (0.18) |
| e) | Ferns | |  |  |  |  |  |  |  |  |  |  |  |
|  |  |  | *Osmunda japonica* | P | Fern | 0.7 | 0.2 | 0.3 | (0.0) | (0.3) |  | 0.3 | (0.0) |
|  |  |  | *Equisetum arvense* | P | Fern | 0.7 | 0.2 | 0.5 | (0.0) | (0.5) |  | 0.8 | (0.0) |
|  |  |  | *Deparia conilii* | P | Fern | 0.4 | 0.0 | 0.0 | (0.0) | (0.0) |  | 0.2 | (0.0) |
|  |  |  | *Deparia japonica* | P | Fern | 0.5 | 0.3 | 0.3 | (0.0) | (0.3) |  | 0.1 | (0.0) |
|  |  |  | *Odontosoria chinensis* | P | Fern | 0.2 | 0.0 | 0.0 | (0.1) | (0.0) |  | 0.3 | (0.0) |
|  |  |  | *Lygodium japonicum* | P | Fern | 1.0 | 0.7 | 0.8 | (0.2) | (0.8) |  | 0.9 | (0.4) |
|  |  | Average | |  |  | 0.58 | 0.23 | 0.32 | (0.05) | (0.32) |  | 0.43 | (0.07) |
| f) | Invasive exotics | | |  |  |  |  |  |  |  |  |  |  |
|  |  |  | *Solidago altissima* | P | Exotic | 0.0 | 0.0 | 0.2 | (0.6) | (0.2) |  | 0.5 | (0.9) |
| g) | Annuals | | |  |  |  |  |  |  |  |  |  |  |
|  |  |  | *Phyllanthus ussuriensis* | A | Small | 0.3 | 0.0 | 0.0 | (0.0) | - |  | 0.0 | (0.0) |
|  |  |  | *Youngia japonica* | A |  | 0.0 | 0.0 | 0.1 | (0.7) | - |  | 0.6 | (0.1) |
|  |  |  | *Erigeron philadelphicus* | A | Exotic | 0.2 | 0.0 | 0.0 | (0.0) | - |  | 0.1 | (0.3) |
|  |  | Average | |  |  | 0.17 | 0.00 | 0.03 | (0.23) | - |  | 0.23 | (0.13) |
| h) | Others | | |  |  |  |  |  |  |  |  |  |  |
|  | h_1_) | Species with frequencies in 2019 and 2020 less than half those in 2008 | | | |  |  |  |  |  |  |  |  |
|  |  |  | *Brachypodium sylvaticum* | P |  | 0.5 | 0.0 | 0.0 | (0.2) | (0.0) |  | 0.2 | (0.3) |
|  |  |  | *Eurya japonica* var. *japonica* | W |  | 0.4 | 0.0 | 0.0 | (0.1) | (0.0) |  | 0.1 | (0.0) |
|  | h_2_) | Species with the lower frequency in 2018 than in 2008, and, at least, either 2019 or 2020 | | | |  |  |  |  |  |  |  |  |
|  |  |  | *Quercus serrata* | W |  | 0.8 | 0.3 | 0.7 | (0.1) | (0.7) |  | 0.8 | (0.0) |
|  |  |  | *Clematis terniflora* | P |  | 0.7 | 0.2 | 0.6 | (0.3) | (0.6) |  | 0.6 | (0.0) |
|  |  |  | *Salvia japonica* | P |  | 0.6 | 0.3 | 0.5 | (0.9) | (0.5) |  | 0.5 | (0.5) |
|  |  |  | *Carex lenta* var. *lenta* | P |  | 0.4 | 0.0 | 0.4 | * | (0.4) |  | 0.4 | (0.1) |
|  |  |  | *Oplismenus undulatifolius* | P |  | 0.6 | 0.0 | 0.0 | (0.1) | (0.0) |  | 0.5 | (0.0) |
|  |  |  | *Broussonetia kaempferi* var. *australis* | W |  | 0.5 | 0.2 | 0.5 | (0.0) | (0.5) |  | 0.7 | (0.0) |
|  |  |  | *Carex lanceolata* | P |  | 0.5 | 0.2 | 0.2 | * | (0.2) |  | 0.3 | (0.0) |
|  |  |  | *Rhododendron kaempferi* var. *kaempferi* | W |  | 0.3 | 0.0 | 0.1 | (0.0) | (0.1) |  | 0.1 | (0.0) |
|  | h_3_) | Species more frequent in 2018, 2019 and 2020 than in 2008, or similar frequency in 2018 to 2008 | | | | |  |  |  |  |  |  |  |
|  |  |  | *Paederia foetida* | P |  | 0.0 | 0.7 | 0.6 | (0.0) | (0.6) |  | 1.0 | (0.0) |
|  |  |  | *Lonicera japonica* | P |  | 0.0 | 0.4 | 0.3 | (0.0) | (0.3) |  | 0.4 | (0.0) |
|  |  |  | *Indigofera pseudotinctoria* | P |  | 0.0 | 0.2 | 0.4 | (0.1) | (0.4) |  | 0.3 | (0.0) |
|  |  |  | *Lilium auratum* | P |  | 0.2 | 0.2 | 0.3 | (0.5) | (0.0) |  | 0.4 | (0.1) |
|  |  |  | *Pueraria lobata* | P |  | 0.0 | 0.1 | 0.6 | (0.0) | (0.6) |  | 0.4 | (0.0) |
|  |  |  | *Dioscorea tokoro* | P |  | 0.0 | 0.0 | 0.2 | (0.0) | (0.2) |  | 0.2 | (0.0) |
|  |  |  | *Rubus palmatus* var. *coptophyllus* | W |  | 0.0 | 0.1 | 0.1 | (0.0) | (0.1) |  | 0.7 | (0.0) |
|  | h_4_) | Species with more or equal frequencies to 0.5 in each year | | |  |  |  |  |  |  |  |  |  |
|  |  |  | *Houttuynia cordata* | P |  | 1.0 | 1.0 | 1.0 | (0.1) | (1) |  | 1.0 | (0.1) |
|  |  |  | *Dioscorea japonica* | P |  | 0.7 | 0.5 | 0.0 | (0.0) | (0.0) |  | 0.7 | (0.0) |
|  |  |  | *Lysimachia clethroides* | P |  | 0.0 | 0.1 | 0.2 | (0.5) | (0.2) |  | 0.1 | (0.4) |
|  |  |  | *Picris hieracioides* subsp. *japonica* | A |  | 0.2 | 0.0 | 0.0 | (0.2) | - |  | 0.1 | (0.5) |
|  |  |  | *Ixeridium dentatum* subsp. *dentatum* | P |  | 0.2 | 0.0 | 0.2 | (0.4) | (0.2) |  | 0.2 | (0.2) |
|  |  |  | *Aphananthe aspera* | W |  | 0.0 | 0.0 | 0.4 | (0.1) | (0.3) |  | 0.0 | (0.0) |
|  |  |  | *Lespedeza pilosa* | P |  | 0.3 | 0.1 | 0.1 | (0.4) | (0.1) |  | 0.2 | (0.1) |
|  |  |  | *Hosta sieboldiana* var. *sieboldiana* | P |  | 0.1 | 0.0 | 0.1 | (0.0) | 0.1) |  | 0.2 | (0.3) |
|  |  |  | *Carex conica* | P |  | 0.2 | 0.0 | 0.3 | (0.0) | (0.3) |  | 0.0 | (0.0) |

A, annual; P, perennial herb; W, woody species. Species with a frequency ≥0.2 in at least 1 year are shown. Increases and decrease were defined when differences in frequency between years were >0.2. Several individuals of *Cirsium* spp. died before identification, so frequencies for *C. oligophyllum* and *C. japonicum* would be underestimated. *All the seedlings of *Carex* ssp. were unidentified to the species level.
