# Supplementary material for: Fast grassland recovery from viable propagules after reintroducing traditional mowing management on a steep slope
Source: PeerJ. 2024 Jun 5;12:e17487. doi: 10.7717/peerj.17487 (PMC11162179; doi:10.7717/peerj.17487)
Supplement: Supplemental Information 3 [file peerj-12-17487-s003.docx]

**Supplementary Table S3** Vegetation cover (%) across the entire study site in May and September in 2019 and 2020

| Year | May | September |
| --- | --- | --- |
| 2019 | 39.0 ± 17.3 | 35.5 ± 10.1 |
| 2020 | 68.5 ± 17.5 | 61.0 ± 11.3 |

Mean values ± SD are shown.
